# Attenuation of opioid tolerance by ET_B_ receptor agonist, IRL-1620, is independent of an accompanied decrease in nerve growth factor in mice

**DOI:** 10.1016/j.heliyon.2017.e00317

**Published:** 2017-06-07

**Authors:** Shruti Gulati, Seema Briyal, Shantel Jones, Shaifali Bhalla, Anil Gulati

**Affiliations:** a Chicago College of Health Sciences, Midwestern University, Downers Grove, IL 60515, USA; b Chicago College of Pharmacy, Midwestern University, Downers Grove, IL 60515, USA

**Keywords:** Neuroscience

## Abstract

**Aim:**

ET_A_ receptor antagonists reverse opioid tolerance but the involvement of ET_B_ receptors is unknown. In morphine or oxycodone tolerant mice we investigated (1) the effect of ET_B_ receptor agonist, IRL-1620, on analgesic tolerance; (2) changes in expression of the brain ET_A_ and ET_B_ receptors; and (3) alterations in the brain VEGF, NGF, PI3K and notch-1 expression.

**Main methods:**

Body weight, body temperature, and tail-flick latency were assessed before and after a challenge dose of morphine or oxycodone in vehicle or IRL-1620 treated mice. Expression studies were carried out using Western blots.

**Key findings:**

Tail flick latency to a challenge dose of opioid was significantly increased by IRL-1620 from 39% to 100% in morphine tolerant and from 8% to 83% in oxycodone tolerant mice. Morphine or oxycodone did not alter ET_A_ or ET_B_ receptor expression. IRL-1620 had no effect on ET_A_ however it increased (61%) expression of ET_B_ receptors. IRL-1620-induced increase in ET_B_ receptor expression was attenuated by morphine (39.8%) and oxycodone (51.8%). VEGF expression was not affected by morphine or oxycodone and was unaltered by IRL-1620. However, NGF and PI3K expression was decreased (P < 0.001) by morphine and oxycodone and was unaffected by IRL-1620. Notch-1 expression was not altered by morphine, oxycodone or IRL-1620.

**Significance:**

ET_B_ receptor agonist, IRL-1620, restored analgesic tolerance to morphine and oxycodone, but it did not affect morphine and oxycodone induced decrease in NGF/PI3K expression. It is concluded that IRL-1620 attenuates opioid tolerance without the involvement of NGF/PI3K pathway.

## 1 Introduction

Opioids such as morphine and oxycodone are among the most potent analgesics used commonly for the management of moderate to severe pain. Due to their high analgesic efficacy, they are considered to be the drug of choice for numerous clinical situations such as managing acute pain following surgery or physical injury and chronic pain due to cancer or arthritis. However, a major limitation of opioid use is the development of rapid tolerance to its analgesic effect, resulting in inadequate pain relief if a higher dose of the drug is not used. There are multiple hypotheses to explain opioid tolerance. One explanation is opioid receptor down-regulation which reduces the number of receptors available for opioid actions [1]. Another explanation is opioid receptor desensitization, where sustained exposure to opioids produces decoupling of the opioid receptors, which leads to signaling desensitization [1, 2]. In general, tolerance mechanisms are extremely complex and not very well understood.

Many drugs that produce tolerance and dependence have been shown to modulate neurogenesis, including methamphetamine [3], cocaine [4] and opioids [5, 6]. Accumulating evidence suggests that opioid drugs have a negative impact on neurogenesis [5, 6, 7, 8, 9]. It was found that morphine decreases the expression of nestin positive cells [7]. Nestin is a neural stem cell marker, which suggests that morphine inhibits self-renewal of neural stem cells [7]. These effects appear to be mediated by opioid receptors since they were reversed with the addition of naloxone, a nonselective opioid receptor antagonist [7]. Neurogenesis is known to persist throughout adult life in the brains of mammals [10, 11, 12]. Chronic administration of morphine markedly decreases neurogenesis in the hippocampus of adult rats [5]. It has been observed that a decrease in opioid agonist analgesic potency is a reflection of decreased neurogenic differentiation 1 (NeuroD1) activity [13]. Further, chronic administration of morphine produced a decrease in NeuroD1 activity and an increase in (effective dose) ED_50_, while chronic administration of fentanyl did not decrease NeuroD1 activity or increase ED_50_. It is known that the ability of fentanyl to induce tolerance is lower than that of morphine [14] further implicating involvement of neurogenesis in the development of opioid tolerance.

Endothelin (ET) has been shown to increase the release of both neurotrophic and angiogenic factors, such as VEGF and NGF, via stimulation of ET_B_ receptors on astrocytes [15, 16]. Since it has been demonstrated that ET_B_ receptors are involved in angiogenesis and neurogenesis in an opposing manner to opioid receptors, it is therefore possible that ET_B_ receptors may be playing a role in opioid tolerance. It has been shown that stimulation of central ET_B_ receptors plays an important role in providing neuroprotection and leads to neurovascular remodeling [12, 17, 18]. Subsequent studies have demonstrated that ET_B_ receptor induced angiogenesis and neurogenesis occurs, at least in part, via altering expression of VEGF, NGF and PI3K [19, 20]. In previous studies, we have shown that ET_A_ receptor antagonists potentiate morphine analgesia in mice and rats [21, 22] and reverse opioid tolerance via a G-protein mediated mechanism [22, 23, 24]. In an acute study it was found that ET_B_ receptors are not involved in morphine analgesia [25], however, the role of ET_B_ receptors in opioid tolerance has never been investigated. Since, both opioid and ET_B_ receptors are involved in neurogenesis, which is being implicated in the development of opioid tolerance, it is convincing to investigate involvement of ET_B_ receptors in opioid tolerance.

The present study was therefore conducted with three aims; (1) we tested the effect of IRL-1620, a selective ET_B_ receptor agonist, on the analgesic tolerance to morphine and oxycodone in mice; (2) we determined the expression of ET_A_ receptors and ET_B_ receptors in the brain of morphine and oxycodone tolerant mice; and (3) we determined the expression of VEGF, NGF, phosphoinositide 3-kinase (PI3K) and notch-1 in the brain of morphine and oxycodone tolerant mice.

## 2 Material and methods

### 2.1 Animals

Male Swiss Webster mice, weighing approximately 25 to 30 g, were used in the present study. Mice were acclimated for a minimum of four days to their environment before experiments were carried out. Animals were housed in a room with controlled temperature (23 ± 1° C), humidity (50 ± 10%), and light (6:00 A.M. to 6:00 P.M.) and food and water were made available *ad libitum*. The Institutional Animal Care and Use Committee (IACUC) of Midwestern University approved all animal use, anesthetics and procedures.

### 2.2 Drugs

Morphine sulfate, 7,8-didehydro-4,5α-epoy-17-methylmorphinan-3,6α-diol sulfate, (Sigma-Aldrich, St. Louis, MO, USA), was dissolved in sterile saline and injected subcutaneously (s.c.). Oxycodone hydrochloride, 4,5α-epoxy-14-hydroxy-3-methoxy-17-methylmorphinan-6-one hydrochloride (Sigma-Aldrich, St. Louis, MO, USA), was dissolved in sterile saline and injected subcutaneously (s.c.). Selective ET_B_ receptor agonist, IRL-1620, (N-Succinyl-[Glu9, Ala11,15]-endothelin 1; Bachem Americas, Inc., Torrance, CA 90505), was dissolved in sterile saline and injected intravenously (i.v.) through the tail vein.

### 2.3 Experimental design and methods

Mice were divided into the following groups: group 1: vehicle (saline, 10 ml/kg, s.c.) + vehicle (saline, 10 ml/kg, i.v.); group 2: opioid agonist (doses/regimen described below) + vehicle (saline, 10 ml/kg, i.v.); group 3: vehicle (saline, 10 ml/kg, s.c.) + IRL-1620 (5 μg/kg, i.v.); group 4: opioid agonist (doses/regimen described below) + IRL-1620 (5 μg/kg, i.v.). Opioid agonist were either morphine or oxycodone. The dose of IRL-1620 was selected based upon previous studies conducted in our laboratory [19, 20].

### 2.4 Induction of tolerance to opioid agonists

**Fig. 1:**
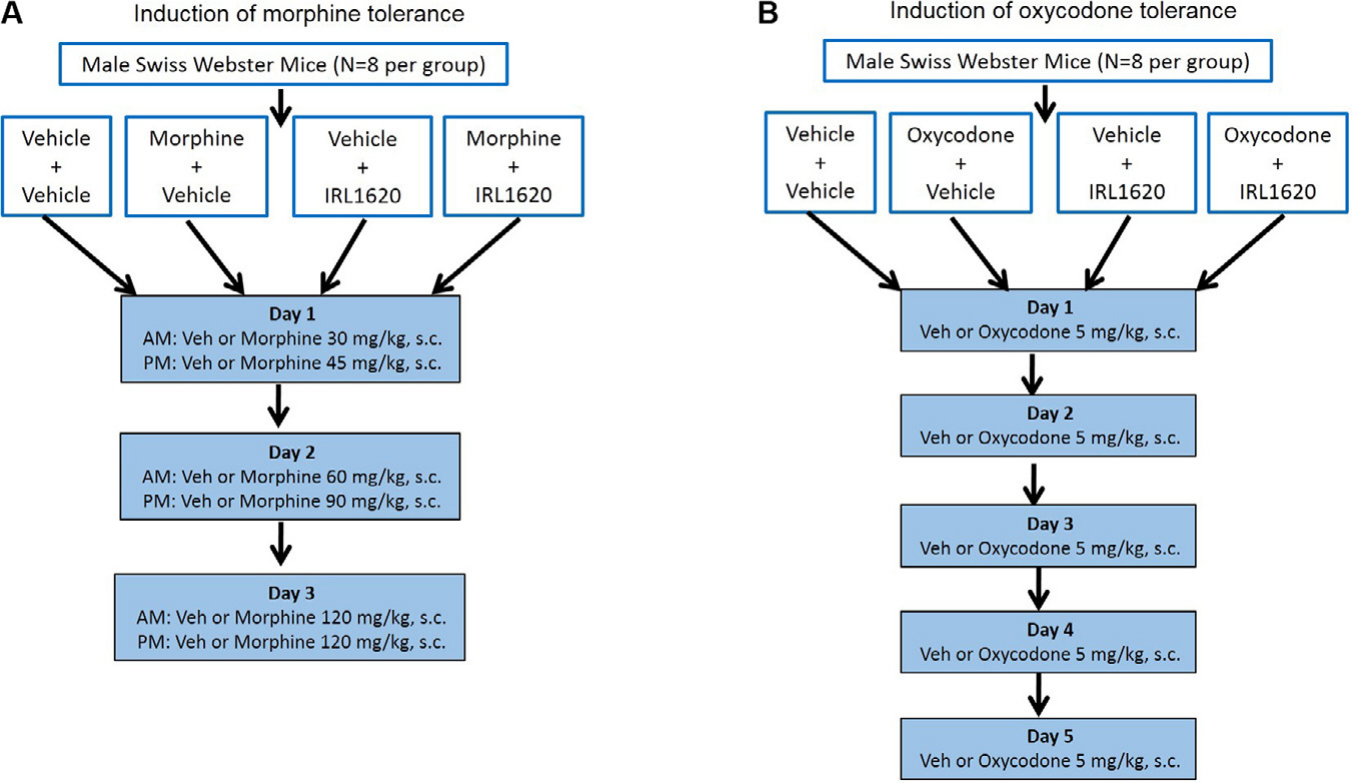
Experimental design and protocol for induction of tolerance to morphine (A) and oxycodone (B) in mice.

Tolerance to morphine was induced using a 3-day cumulative dosing regimen [26, 27]. Morphine treatment schedule consisted of twice-daily s.c. injections of morphine for three days given at (i) 30 mg/kg (a.m.) and 45 mg/kg (p.m.) on day 1; (ii) 60 mg/kg (a.m.) and 90 mg/kg (p.m.) on day 2; and (iii) 120 mg/kg twice (a.m. and p.m.) on day 3 (Fig. 1A). The IRL-1620 treatment schedule consisted of three times-daily injections of IRL-1620 for two days given at 5 μg/kg, i.v. spaced apart every 2 h on days 1 and 3. At the end of the treatment schedule, a challenge dose of morphine (5 mg/kg, s.c.) was administered on day 4 to assess tolerance.

Tolerance to oxycodone was induced using a 5-day cumulative dosing regimen [24, 27]. Oxycodone treatment schedule consisted of once-daily injections of 5 mg/kg, s.c. of oxycodone for five days (Fig. 1B). The IRL-1620 treatment schedule consisted of three times-daily injections of IRL-1620 for three days given at 5 μg/kg, i.v. spaced apart every 2 h on days 1, 3 and 5. At the end of the treatment schedule, a challenge dose of oxycodone (5 mg/kg, s.c.) was administered on day 6 to assess tolerance.

Body temperature and tail flick latency were determined before treatment (baseline) and at 30, 60, 90, 120, 180, 240, 300, and 360 min. post administration of morphine or oxycodone challenge dose. Tolerance was assessed based on loss of antinociceptive effect of morphine or oxycodone using the tail-flick latency assay. Body weight was determined at the beginning of each day.

### 2.5 Measurement of body temperature

The rectal temperature of each mouse was recorded before (at baseline) and at various times (30, 60, 90, 120, 180, 240, 300 and 360 min) after the injection of morphine and oxycodone challenge dose, using lubricated Cole Palmer Animal Monitoring Thermometer with colonic probe (Vernon Hills, IL). Mice were not restrained and the thermometer was only inserted at the specific time points in order to reduce stress induced variables. Body temperature in degrees Celsius (°C) was plotted versus time and expressed as mean ± S.E.M.

### 2.6 Measurement of tail-flick latency

Antinociceptive responses to morphine and oxycodone were determined by the tail-flick latency method [28]. The tail-flick latency assay measures analgesic responses modulated by the spinal cord [29, 30]. Application of thermal radiation (by focused light) was done on a blackened spot 1–2 cm from the tip of the tail of the animal, provoking withdrawal of the tail by a brief vigorous movement. The withdrawal time was recorded as tail-flick latency using an analgesiometer (Panlab, LE 7106 Light Beam Analgesy-Meter, at an infrared intensity of 50). Tail-flick latencies to thermal stimulation were determined at baseline (before any drug administration), and at 30, 60, 90, 120, 180, 240, 300 and 360 min after injection of the challenge dose of morphine or oxycodone. A cutoff time of 10 s was used to prevent damage to the tail. Antinociceptive responses in each treatment group were expressed as mean ± S.E.M.

### 2.7 Estimation of endothelin receptors, growth factors, and signaling pathways

Expression of ET_A_ and ET_B_ receptors, NGF, VEGF, PI3K, and notch-1 was determined using the Western blotting technique [19, 20, 31] with some modifications. After completion of behavioral experiments animals were sacrificed and brains were immediately dissected out, flash frozen on dry ice, and stored at −80 °C for further analysis. The tissue was homogenized with 10x (w/v) RIPA lysis buffer (20 mM Tris-HCl pH 7.5, 120 mM NaCl, 1.0% TritonX-100, 1.0% sodium deoxycholate, 0.1% sodium dodecyl sulfate (SDS), 10% glycerol, 1 mM disodium ethylene diamine tetraacetic acid (EDTA), 1 mM ethylene glycol-bis(β −aminoethyl ether)-N,N,N’,N’-tetraacetic acid tetrasodium salt (EGTA), phosphatase inhibitors and Complete Mini Protease inhibitor cocktail tablet (Roche Diagnostics, Indianapolis, IN). Proteins were isolated in solubilized form and concentrations were measured by Folin-Ciocalteu’s phenol reagent [32]. Solubilized protein (60 μg) was denatured in Laemmli sample buffer (Bio-Rad Laboratories, Hercules, CA), resolved on 10% sodium dodecyl sulfate-polyacrylamide gel electrophoresis (SDS-PAGE) and then transferred onto the nitrocellulose membrane followed by blocking the membrane with SuperBlock^®^ Blocking Buffer in tris-buffered saline (TBS) (ThermoFisher Scientific, Hanover Park, IL). The membranes were washed three times with 1x TBS-Tween (TBST) and incubated with rabbit polyclonal anti-ET_A_ receptor (ab85163, Abcam, Cambridge, MA, 1:1000) or anti-ET_B_ receptor (ab117529, Abcam, Cambridge, MA, 1:1000) or anti-VEGF or anti-NGF (ab46154 and ab52918, Abcam, Cambridge, MA, 1:1000) or monoclonal anti-PI3K (4257, Cell Signaling Technology, Danvers, MA, 1:1000) or anti-notch1 (ab52627, Abcam, Cambridge, MA, 1:1000) or mouse anti-β-tubulin (sc55529, Santa Cruz Biotechnology, Dallas, TX) or mouse monoclonal anti-β-actin (a1978, Sigma-Aldrich, St. Louis, MO) antibodies, followed by horseradish peroxidase (HRP)-conjugated secondary antibodies goat anti-rabbit (sc2004, Santa Cruz Biotechnology, Dallas, TX, 1:2000) or goat anti-mouse (ab98693, Abcam, Cambridge, MA, 1:10,000) and visualized by SuperSignal^®^ West Pico Chemiluminescent Substrate (ThermoFisher Scientific, Hanover Park, IL) using the ChemiDoc™ MP Imaging System (Bio-Rad Laboratories, Hercules, CA) and then analyzed using ImageJ (NIH) software.

### 2.8 Statistics

A Power Analysis was conducted using GraphPad Instat-3.1. The power was set to 80% (beta = 0.8) and the level of significance (alpha) used was 0.05. The sample size in each group was N = 8 in behavioral and N = 4 in Western blot experiments based upon expected change determined from results published in literature using similar procedures. Percent change in tail-flick latency in response to the morphine or oxycodone challenge dose was calculated as 100% x [(Drug Response Time − Basal Response Time)/(Basal Response Time)]. The AUC was calculated to depict the magnitude and duration of antinociceptive response to better express the degree of antinociceptive tolerance. The baseline values were subtracted from the values obtained on the last day of the experimental protocol (day 4 for morphine treated group; day 6 for the oxycodone treated group) to determine area under the curve (AUC) for tail flick latency. The values are presented as Mean ± S.E.M. (Standard Error of Mean). The AUC for each treatment group was determined using the trapezoidal rule, and the difference in the AUC of each treatment compared to control treatments were calculated. In the trapezoidal rule, the area of each trapezoid is equal to the product of the height at the midpoint (on the vertical axis) and the width of the base (horizontal axis). The total area is calculated by adding areas of a series of trapezoids using Microsoft Excel 2016 program [33].

Statistical differences within and between groups were analyzed by a two-way repeated measures ANOVA test using GraphPad Prism version 7.00 for Windows (GraphPad software, San Diego, CA, USA). Following that, a one-way analysis of variance (ANOVA) followed by post-hoc test (Tukey’s multiple comparisons test) was conducted. A level of P < 0.05 was considered significant.

## 3 Results

### 3.1 Effect of ET_B_ receptor agonist, IRL-1620, on body weight of mice tolerant to morphine and oxycodone

**Fig. 2:**
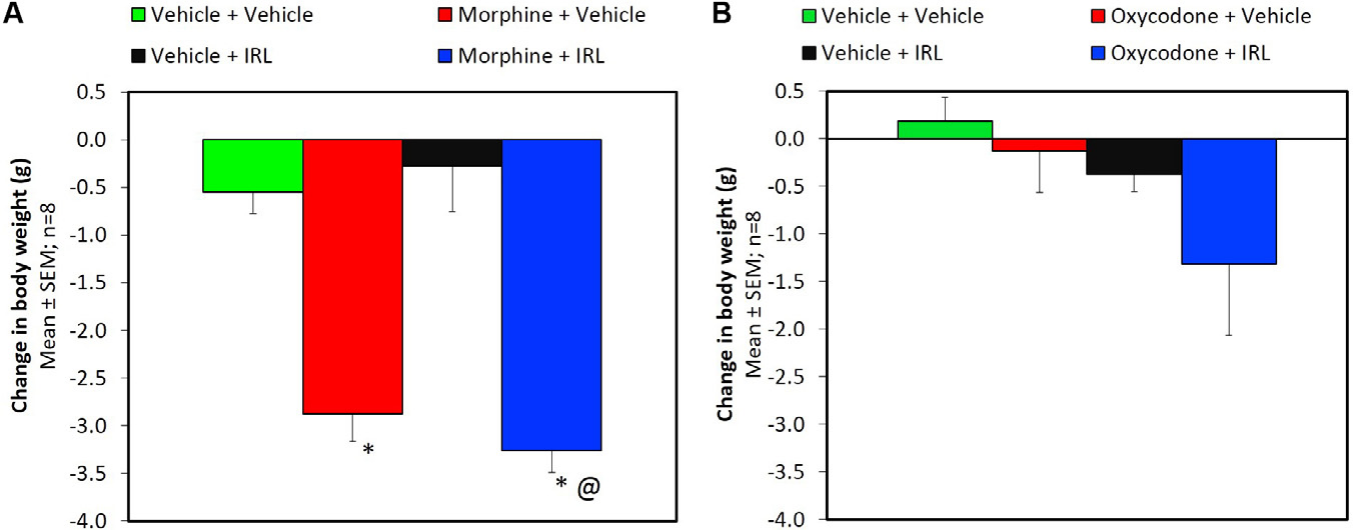
Change in body weight of mice during development of tolerance to morphine (A) and oxycodone (B) and influence of IRL-1620 on those changes. *p < 0.05 vs. Vehicle + Vehicle; @p < 0.05 vs. Vehicle + IRL-1620.

The change in body weight measured at baseline and post tolerance is presented in Fig. 2. The decrease in body weight in the morphine + vehicle group was significantly (P < 0.01) greater than that observed in the vehicle + vehicle group. The vehicle + IRL-1620 group showed a decrease in body weight. However, a decrease in body weight in morphine + IRL-1620 group was similar to that observed in morphine + vehicle group (Fig. 2A). These findings indicate a loss in body weight in mice undergoing morphine tolerance is not altered by IRL-1620. On the other hand, in oxycodone group (Fig. 2B) there was no change in body weight in any group, indicating that oxycodone tolerance did not have any effect on the body weight.

### 3.2 Effect of ET_B_ receptor agonist, IRL-1620, on morphine and oxycodone induced changes in body temperature of mice rendered tolerant to morphine and oxycodone

**Fig. 3:**
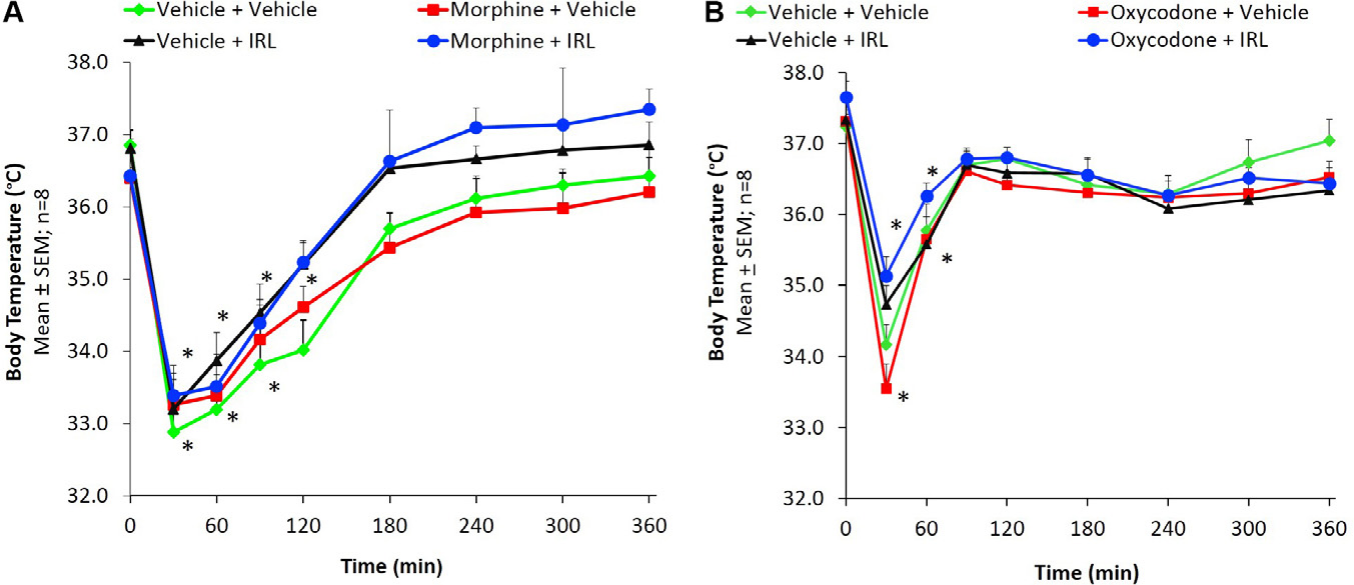
Change in body temperature of mice during development of tolerance to morphine (A) and oxycodone (B) and influence of IRL-1620 on those changes. *p < 0.05 compared to baseline.

In morphine tolerance studies a challenge dose of morphine produced hypothermia and the maximum effect was observed at 30 min. Baseline body temperature in the control (vehicle + vehicle) group was 36.85 ± 0.21 °C and significantly (P < 0.0001) dropped to 32.88 ± 0.26 °C after a challenge dose of morphine (Fig. 3A). A challenge dose of morphine produced similar hypothermic effect in morphine + vehicle, vehicle + IRL-1620 and morphine + IRL-1620 groups (Fig. 3A). In the oxycodone study, a challenge dose of oxycodone produced hypothermia and the maximum effect was observed at 30 min. Baseline body temperature in the control (vehicle + vehicle) group was 37.23 ± 0.18 °C and significantly (P < 0.0001) decreased to 34.17 ± 0.28 °C after oxycodone challenge dose (Fig. 3B). A challenge dose of oxycodone produced similar hypothermic effect in oxycodone + vehicle, vehicle + IRL-1620 and oxycodone + IRL-1620 groups (Fig. 3B). These findings indicate that tolerance did not develop to morphine- or oxycodone-induced hypothermic effect and was not affected by IRL-1620.

### 3.3 Effect of ET_B_ receptor agonist, IRL-1620, on morphine and oxycodone induced changes in tail-flick latency of mice rendered tolerant to morphine and oxycodone

**Fig. 4:**
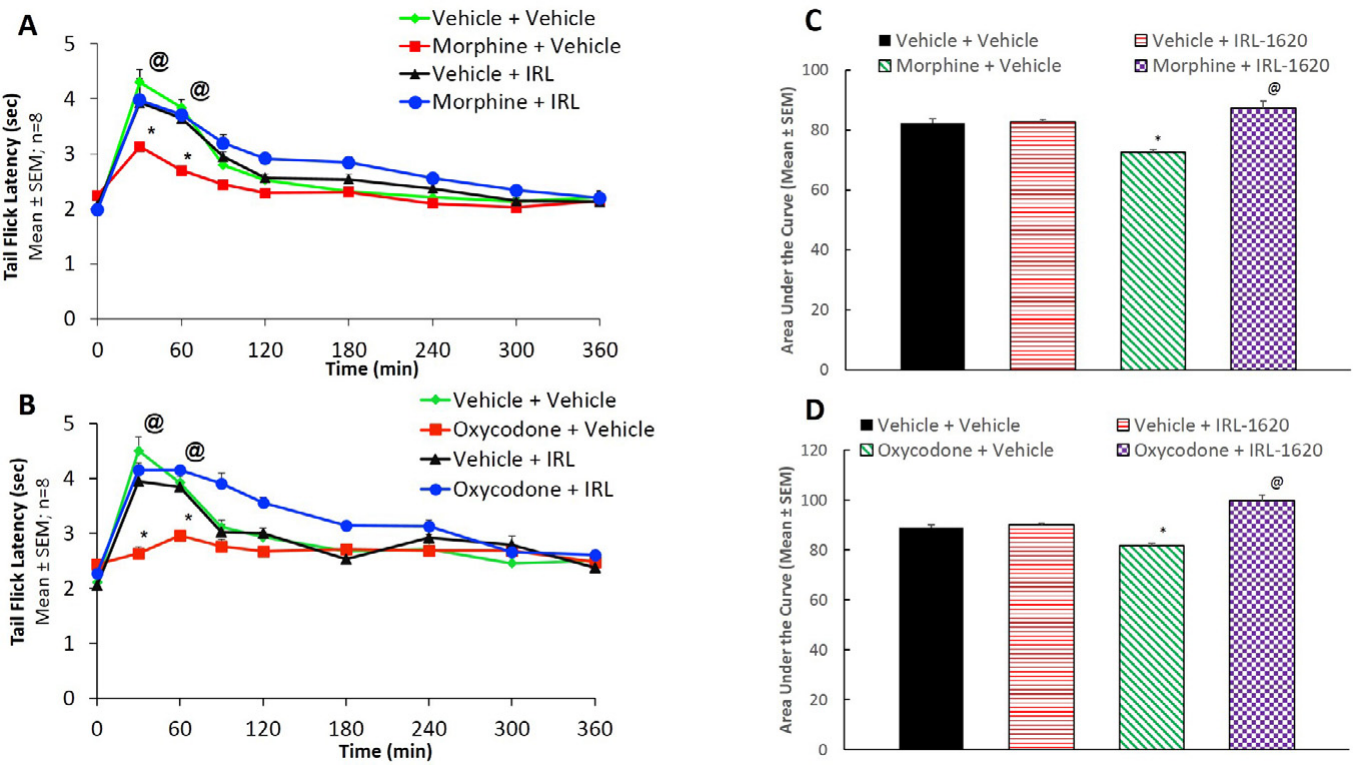
Change in tail-flick latency of mice during development of tolerance to morphine (A) or oxycodone (B). Area under the curve for morphine (C) or oxycodone (D) is provided as bar diagrams. *p < 0.05 vs. Vehicle + Vehicle; @p < 0.05 vs. Opioid + Vehicle.

Baseline tail-flick latency without any drug treatment in the control (vehicle + vehicle) group was 1.98 ± 0.10 s in morphine and 2.12 ± 0.18 s in oxycodone groups. In all other groups the baseline tail-flick latency values were similar to that observed in the control group (Fig. 4). In morphine tolerance studies a challenge dose of morphine produced an increase in tail-flick latency with maximal effect at 30 min (Fig. 4A). In the control (vehicle + vehicle) group tail-flick latency increased (117%; P < 0.0001) in response to a challenge dose of morphine. A similar response was obtained in vehicle + IRL-1620 group. In vehicle-treated morphine tolerant mice, the baseline tail-flick latency was 2.25 ± 0.06 s and a challenge dose of morphine produced only 39% increase latency indicating significant (P < 0.01) development of tolerance (Fig. 4A). However, in IRL-1620-treated morphine tolerant mice a significant (100%; P < 0.0001) increase in antinociceptive response to a challenge dose of morphine was observed (Fig. 4A). In oxycodone tolerant mice a challenge dose of oxycodone resulted in significant (P < 0.0001) potentiation of antinociceptive response to 3.67 ± 0.25 s (Fig. 4B). A similar response was obtained in the IRL-1620 treated group (Fig. 4B). In vehicle treated oxycodone tolerant mice the baseline tail-flick latency was increased only by 8% in response to a challenge dose of oxycodone. However, in IRL-1620 treated oxycodone tolerant mice the antinociceptive response significantly (P < 0.0001) increased by 83% to oxycodone challenge dose (Fig. 4B). The results indicate that IRL-1620 significantly reduced the development of tolerance to morphine and oxycodone (Fig. 4). Details of statistical analysis and values of F and degrees of freedom are shown in Table 1.

**Table 1:** A two-way repeated measures ANOVA test has been conducted using GraphPad Prism version 7.00 for Windows (GraphPad software, San Diego, CA, USA). Values of F and degrees of freedom are shown for morphine in Table 1A and for oxycodone in Table 1B.

TABLE 1A	Morphine Tail-Flick Latency				
	**Two-way ANOVA with repeated measures**				
Alpha	0.05				
**Source of Variation**	**% of total variation**	**P value**	**P value summary**	**Significant?**	
Interaction	7.48	<0.0001	****	Yes	
Time	67.2	<0.0001	****	Yes	
Treatment group	6.039	<0.0001	****	Yes	
**ANOVA table**	**SS**	**DF**	**MS**	**F (DFn, DFd)**	**P value**
Interaction	10.94	24	0.456	F (24, 252) = 4.073	P < 0.0001
Time	98.31	8	12.29	F (8, 252) = 109.8	P < 0.0001
Treatment group	8.836	3	2.945	F (3, 252) = 26.31	P < 0.0001
Residual	28.21	252	0.112		

**TABLE 1B**	**Oxycodone Tail-Flick Latency**				

	**Two-way ANOVA with repeated measures**				
Alpha	0.05				
**Source of Variation**	**% of total variation**	**P value**	**P value summary**	**Significant?**	
Interaction	17.54	<0.0001	****	Yes	
Time	51.68	<0.0001	****	Yes	
Treatment group	7.15	<0.0001	****	Yes	
**ANOVA table**	**SS**	**DF**	**MS**	**F (DFn, DFd)**	**P value**
Interaction	24.56	24	1.024	F (24, 252) = 7.797	P < 0.0001
Time	72.37	8	9.046	F (8, 252) = 68.92	P < 0.0001
Treatment group	10.01	3	3.338	F (3, 252) = 25.43	P < 0.0001
Residual	33.08	252	0.1313		

### 3.4 Effect of ET_B_ receptor agonist IRL-1620 on alteration in ET_A_ and ET_B_ receptors, NGF, VEGF, PI3K and notch-1 expression in the brain of mice tolerant to morphine and oxycodone

**Fig. 5:**
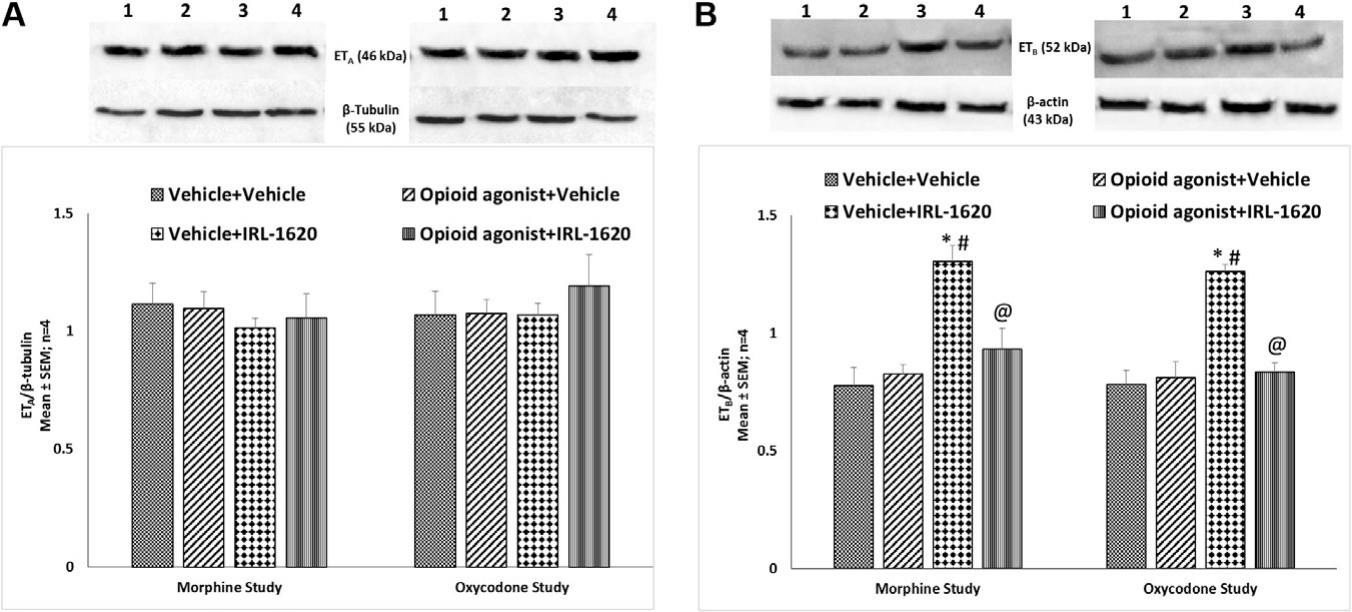
Change in the expression of ET_A_ (A) and ET_B_ (B) receptors in the brain of mice during development of tolerance to opioid agonist morphine or oxycodone and influence of IRL-1620 on those changes. Lane 1- Vehicle + Vehicle; Lane 2 − Opioid agonist + Vehicle; Lane 3 − Vehicle + IRL-1620; Lane 4 − Opioid agonist + IRL-1620. Values are expressed as mean + S.E.M. *p < 0.001 vs. Vehicle + Vehicle; #p < 0.05 vs. Opioid agonist + Vehicle; @p < 0.001 vs. Vehicle + IRL-1620. Full, uncropped images have been provided as supplementary Fig. 5A and 5B.

**Fig. 6:**
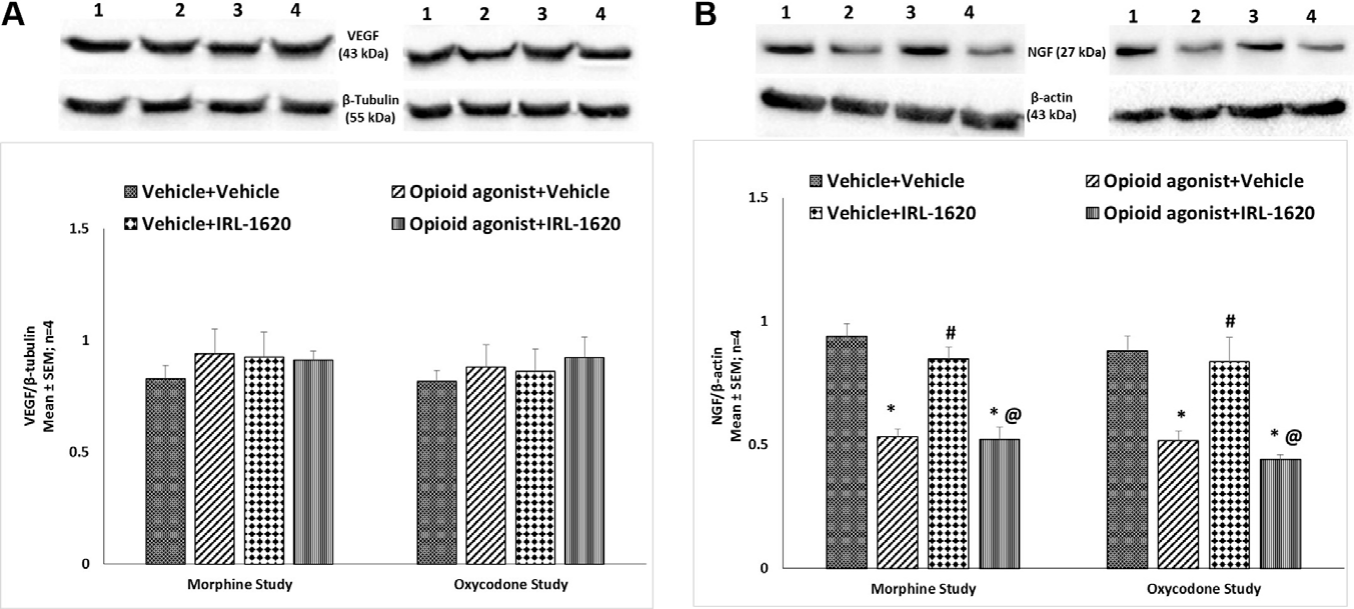
Change in the expression of VEGF (A) and NGF (B) in the brain of mice during development of tolerance to opioid agonist morphine or oxycodone and influence of IRL-1620 on those changes. Lane 1- Vehicle + Vehicle; Lane 2 − Opioid agonist + Vehicle; Lane 3 − Vehicle + IRL-1620; Lane 4 − Opioid agonist + IRL-1620. Values are expressed as mean + S.E.M. *p < 0.001 vs. Vehicle + Vehicle; #p < 0.05 vs. Opioid agonist + Vehicle; @p < 0.001 vs. Vehicle + IRL-1620. Full, uncropped images have been provided as supplementary Fig. 6A and 6B.

**Fig. 7:**
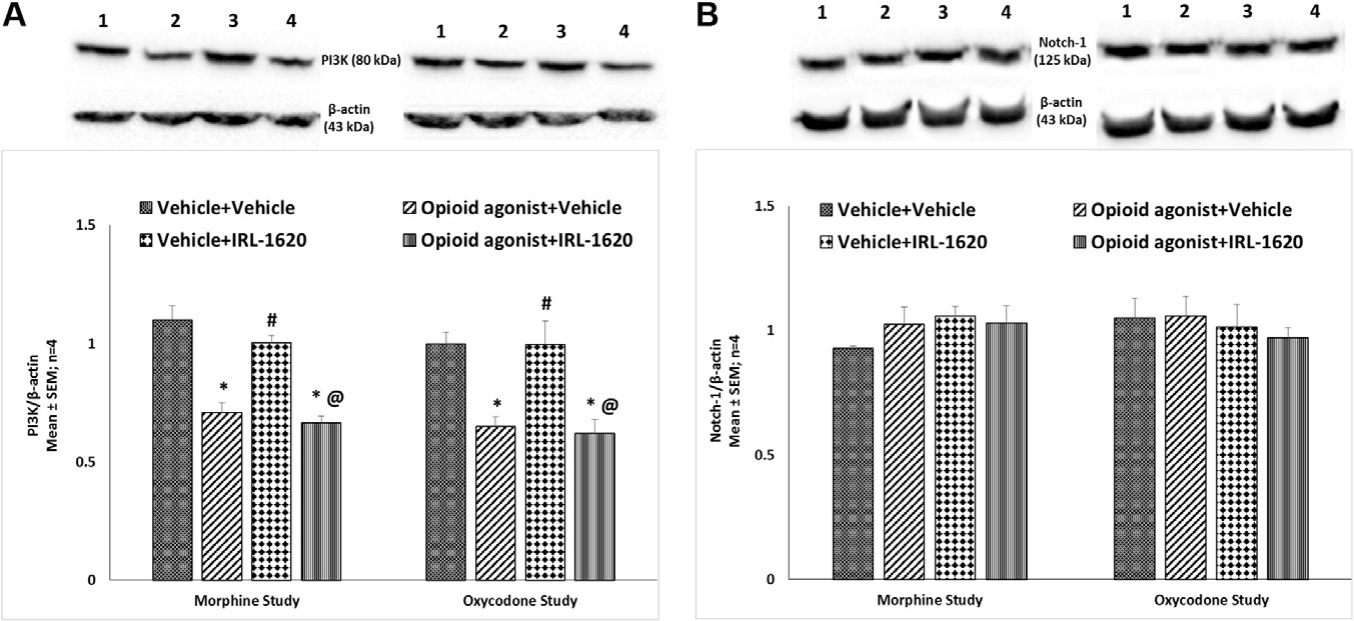
Change in the expression of PI3K (A) and notch-1 (B) in the brain of mice during development of tolerance to opioid agonist morphine or oxycodone and influence of IRL-1620 on those changes. Lane 1–Vehicle + Vehicle; Lane 2–Opioid agonist + Vehicle; Lane 3–Vehicle + IRL-1620; Lane 4–Opioid agonist + IRL-1620. Values are expressed as mean + S.E.M. *p < 0.001 vs. Vehicle + Vehicle; #p < 0.05 vs. Opioid agonist + Vehicle; @p < 0.001 vs. Vehicle + IRL-1620. Full, uncropped images have been provided as supplementary Fig. 7A and 7B.

ET_A_ and ET_B_ receptors, NGF, VEGF, PI3K and notch-1 protein expression levels were examined in the whole brain of mice tolerant to morphine or oxycodone. There was no change in the expression of ET_A_ receptors in morphine or oxycodone tolerant mice, with or without IRL-1620 treatment (Fig. 5A). A significant up-regulation of ET_B_ receptor expression was observed in the brain of control mice treated with the ET_B_ receptor agonist, IRL-1620 (P < 0.05) (Fig. 5B). However, in morphine and oxycodone tolerant mice IRL-1620 induced up-regulation of ET_B_ receptors was not observed indicating that IRL-1620 induced increase in ET_B_ receptor expression is attenuated by chronic treatment with morphine or oxycodone (Fig. 5B). No change in VEGF levels was observed in mice tolerant to morphine or oxycodone, and treatment of IRL-1620 did not alter VEGF expression (Fig. 6A). On the other hand, a significant (P < 0.05) decrease in the expression of NGF was observed in both morphine and oxycodone tolerant mice compared to the control group (Fig. 6B). IRL-1620 alone did not produce any change in the expression of NGF. However, a significant (P < 0.05) decrease in the expression of NGF was observed in morphine and oxycodone tolerant mice treated with IRL-1620, and NGF expression was similar to that observed in vehicle treated morphine and oxycodone tolerant mice (Fig. 6B). A significant (P < 0.05) decrease in the expression of PI3K was observed in both morphine and oxycodone tolerant mice compared to the control treated mice (Fig. 7A). IRL-1620 treatment did not produce any change in the expression of PI3K. However, there was a significant (P < 0.001) decrease in the expression of PI3K with morphine + IRL-1620 and oxycodone + IRL-1620 treatment compared to the control treatment (Fig. 7A). Changes in the expression of PI3K mirrored those of NGF. No change in notch-1 levels were observed in mice that had undergone morphine or oxycodone tolerance, with or without the treatment of IRL-1620 (Fig. 7B).

## 4 Discussion

This study examined the role of ET_B_ receptor stimulation by IRL-1620 in morphine and oxycodone tolerant mice. Results provide evidence for the first time that ET_B_ receptor agonist, IRL-1620, attenuates the development of tolerance to morphine and oxycodone. We also determined possible mechanism and found that opioid tolerance decreased the expression of NGF and its signaling pathway PI3K, though, VEGF expression was not affected. However, IRL-1620 did not attenuate opioid induced decrease in NGF/PI3K pathway, suggesting that attenuation of opioid tolerance by IRL-1620 is not mediated through NGF/PI3K pathway.

Morphine tolerance produced a significant decrease in body weight, while it was not affected by oxycodone tolerance. IRL-1620 did not produce any effect on morphine induced change in body weight. A challenge dose of morphine and oxycodone in tolerant mice produced a significant decrease in body temperature, which was not altered by IRL-1620. Tail-flick latencies in morphine and oxycodone tolerant mice were decreased in response to their challenge dose, however, IRL-1620 significantly increased tail-flick latencies in both morphine and oxycodone tolerant mice. These results provide clear evidence that the development of analgesic tolerance to morphine and oxycodone is attenuated by IRL-1620, because the antinociceptive effect of morphine and oxycodone in tolerant mice was similar to that observed in non-tolerant mice. It can be interpreted that ET_B_ receptor agonists may be effectively used in combination with opioids to prevent the development of tolerance and possibly avoid long-term adverse effects associated with opioids such as tolerance and dependence.

Previous studies have shown that ET_A_ receptors are involved in opioid analgesia [21, 22, 23, 24]. Therefore, in the present study, we sought to determine changes in the expression of ET_A_ receptors in opioid tolerance. No change was observed in the expression of ET_A_ receptors in the brains of mice that underwent morphine or oxycodone tolerance, with or without IRL-1620 treatment. It can be interpreted that development of opioid tolerance does not affect the expression of brain ET_A_ receptors. We subsequently determined changes in the expression of ET_B_ receptors in opioid tolerance. Treatment with IRL-1620, produced a significant up-regulation of ET_B_ receptors in vehicle treated mice but not in morphine and oxycodone tolerant mice. This finding indicates that the up-regulation of ET_B_ receptors due to IRL-1620 was attenuated by morphine or oxycodone tolerance. It is possible that the challenge dose of morphine or oxycodone either alone or when administered to animals pretreated with ET_B_ receptor agonist, IRL-1620, caused an increase in ET_B_ receptor expression and because of opioid tolerance this increase was not observed in IRL-1620 treated mice. However, this explanation is less likely because a challenge dose of morphine or oxycodone was administered to each animal including those in the vehicle treated groups that were or were not tolerant to opioid and no change in the expression of ET_B_ receptors was observed.

Serum levels of NGF were increased in patients suffering from heroin withdrawal, while VEGF levels were not altered [34]. It was also demonstrated that endogenous opioids such as Met-enkephalin decrease angiogenesis and both the number of blood vessels and the total length of vessels are significantly decreased [35]. These findings prompted us to examine the expression of VEGF in the brains of mice following morphine or oxycodone tolerance. VEGF levels did not change following morphine or oxycodone tolerance, with or without treatment of IRL-1620.

Accumulating evidence suggests that the NGF family of neurotrophins have important modulatory roles in opioid analgesia and addiction [36]. The NGF family of neurotrophins include NGF, brain derived neurotrophic factor (BDNF), neurotrophin-3 (NT3), and neurotrophin-4 (NT4) [37]. BDNF knockout mice demonstrated reduction in the intensity of morphine withdrawal symptoms [38]. Intrathecal administration of NGF restored the effectiveness of morphine in attenuating hyperalgesia following peripheral nerve injury [39]. Additionally, a marked attenuation of morphine tolerance was observed in NT4 deficient transgenic mice, indicating that neurotrophin activity is involved in opioid tolerance and dependence [40]. In the present study we found that there was a significant decrease in NGF expression in the brain of mice that had undergone morphine or oxycodone tolerance. A similar decrease in NGF expression was also observed in mice that had undergone morphine or oxycodone tolerance with IRL-1620 treatment. Our results showing that repeated subcutaneous morphine or oxycodone injections decrease NGF and possibly neurogenesis, confirm the findings of a previous report that first observed a decrease in the expression of NGF in both morphine pellet implanted and heroin self-administering rats [5].

Since we found that expression of NGF is significantly decreased in opioid tolerance, and previously we have demonstrated that IRL-1620 increases VEGF and NGF expression in the infarcted hemisphere of the rat with cerebral ischemia [19], we wanted to examine the signaling pathway that involves both ET_B_ receptors and NGF. One signaling pathway that is common to both ET_B_ receptors and NGF is phosphoinositide 3-kinase (PI3K) pathway [41, 42]. Hence, we examined the expression of PI3K in the brains of mice following morphine or oxycodone tolerance. IRL-1620 did not produce any change in the expression of PI3K following morphine or oxycodone tolerance. However, we found that in mice that had undergone morphine or oxycodone tolerance, there was a significant decrease in the expression of PI3K in the brain. A similar decrease in the expression of PI3K was also observed in mice that had undergone morphine or oxycodone tolerance with IRL-1620 treatment. Overall, the data for the expression of PI3K is very similar to the data for the expression of NGF. Alteration in PI3K are a mirror image of NGF expression changes, suggesting that PI3K is involved in the signaling pathway of NGF.

In contrast to our expectation, although IRL-1620 attenuated analgesic tolerance to morphine and oxycodone, but did not produce any change in the expression of NGF. IRL-1620 has been found to enhance [19, 20, 43] the neurorestorative processes that are endogenously induced in brain tissue in response to injury to the brain [44, 45]. Chronic exposure to drugs of abuse produces adaptive changes in various neurotrophic factors in the ventral tegmental neurons and other neurons of the brain [38, 46]. In the present study, there is evidence of adaptive change in the brain of mice that underwent morphine or oxycodone tolerance as indicated by a decrease in NGF expression following morphine or oxycodone tolerance. However, it is possible that adaptive change due to morphine or oxycodone tolerance did not initiate ET_B_ receptor-mediated neuroprotective processes. The adaptive changes due to morphine or oxycodone tolerance are considered to be a reversible process [47] and may be different from those due to neurodegenerative diseases or traumatic brain injuries. This may explain why IRL-1620 did not produce angiogenesis and neurogenesis in morphine or oxycodone tolerance as observed in animal models of cerebral ischemia and Alzheimer’s disease [12, 19, 20, 43].

The notch signaling pathway is a highly conserved pathway that regulates neurogenesis [48, 49]. It is critical in the maintenance, proliferation, differentiation and apoptosis of neuronal stem cells [50, 51]. Notch expression in the sub-ventricular zone of the brain is decreased with aging and co-relates with reduced neurogenesis [52]. Blockage of notch signaling either by siRNA against notch reduced ischemia-mediated cell proliferation, and down-regulation of notch expression leads to an increase in neuronal proliferation [53]. Furthermore, it has been recently shown that autophagy degrades notch1 in primary neurons and induces neuronal stem cell proliferation and differentiation [54]. Neuronal damage results in disruption of the notch signaling pathway, which subsequently leads to neurogenesis [48, 49]. In summary these studies indicate that down-regulation of notch1 leads to increased neurogenesis and up-regulation of notch1 produces decreased neurogenesis indicating the importance of exploring notch signaling in opioid induced alteration in neurogenesis.

We examined the expression of notch-1 in the brains of mice following morphine or oxycodone tolerance. The results showed that notch-1 levels did not change following morphine or oxycodone tolerance, with or without the treatment of IRL-1620. This indicates the possibility that morphine or oxycodone tolerance did not produce any brain damage to induce neurogenesis. We did experiments in the rat brains with right sided cerebral ischemia, where IRL-1620 has been shown to induce neurogenesis and angiogenesis [19], and found a massive decrease in the expression of notch-1 in the right cerebral hemisphere compared to non-ischemic left cerebral hemisphere (data not shown). This suggests that morphine and oxycodone tolerance produces adaptive changes in the brain that are different from those produced by neurodegenerative diseases.

In summary, to our knowledge, this the first study indicating that ET_B_ receptor agonist, IRL-1620 attenuated the development of analgesic tolerance to morphine and oxycodone. We investigated the possibility of the involvement of VEGF and NGF in morphine or oxycodone tolerance and found that there was a significant decrease in the expression of NGF in the brains of the mice following morphine or oxycodone tolerance. However, whether these changes mediate opioid tolerance is not clear. Similar findings were obtained in the expression of PI3K which has been implicated in the signaling pathway of NGF. Additionally, it was found that the expression of notch-1 did not change following morphine or oxycodone tolerance, with or without the treatment of IRL-1620, indicating to the possibility that morphine or oxycodone tolerance did not produce enough damage to the brain to induce the neurorestorative and neuroprotective processes.

## 5 Conclusion

IRL-1620, an ET_B_ receptor agonist, attenuated tolerance to morphine and oxycodone induced analgesia. A significant decrease in the expression of NGF/PI3K in the brain was observed following opioid tolerance which was not affected by ET_B_ receptor agonist, IRL-1620. It is concluded that ET_B_ receptor agonist, IRL-1620 attenuates tolerance to opioids without the involvement of NGF/PI3K pathway.

## Declarations

### Author contribution statement

Shruti Gulati: Conceived and designed the experiments; Performed the experiments; Analyzed and interpreted the data; Contributed reagents, materials, analysis tools or data; Wrote the paper.

Seema Briyal: Performed the experiments; Analyzed and interpreted the data; Contributed reagents, materials, analysis tools or data.

Shantel Jones: Performed the experiments; Contributed reagents, materials, analysis tools or data.

Shaifali Bhalla: Conceived and designed the experiments; Performed the experiments; Analyzed and interpreted the data.

Anil Gulati: Conceived and designed the experiments; Analyzed and interpreted the data; Wrote the paper.

### Funding statement

This work was supported by Midwestern University.

### Competing interest statement

The authors declare no conflict of interest.

### Additional information

No additional information is available for this paper.

## Appendix A Supplementary data

Supplementary Fig. 5A

Supplementary Fig. 5B

Supplementary Fig. 6A

Supplementary Fig. 6B

Supplementary Fig. 7A

Supplementary Fig. 7B
