# Supplementary figures and images for: Attenuation of opioid tolerance by ETB receptor agonist, IRL-1620, is independent of an accompanied decrease in nerve growth factor in mice
Source: Heliyon. 2017 Jun 7;3(6):e00317. doi: 10.1016/j.heliyon.2017.e00317 (PMC5466593; doi:10.1016/j.heliyon.2017.e00317)

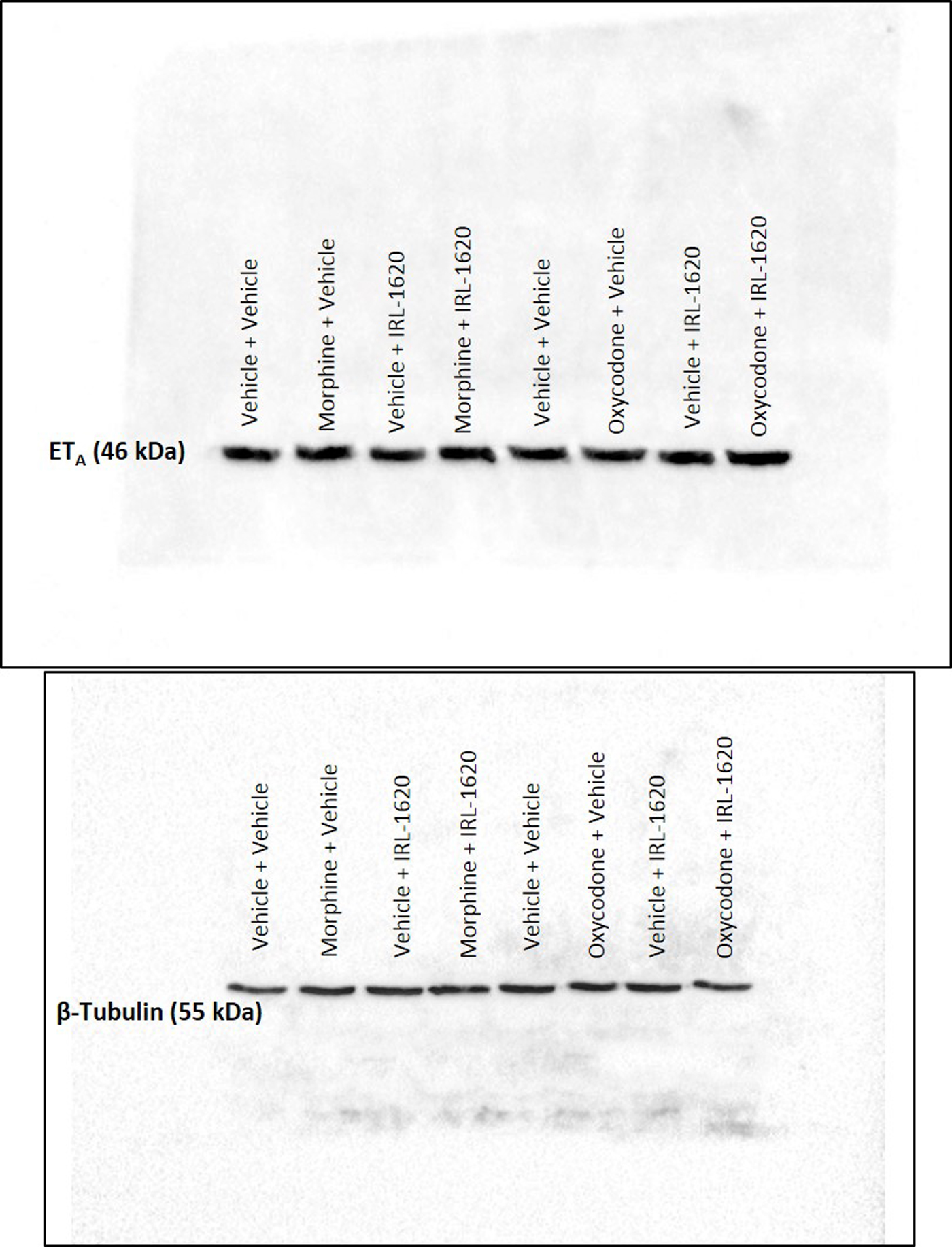

Supplement: Supplementary Fig. 5A [file mmc1.jpg]

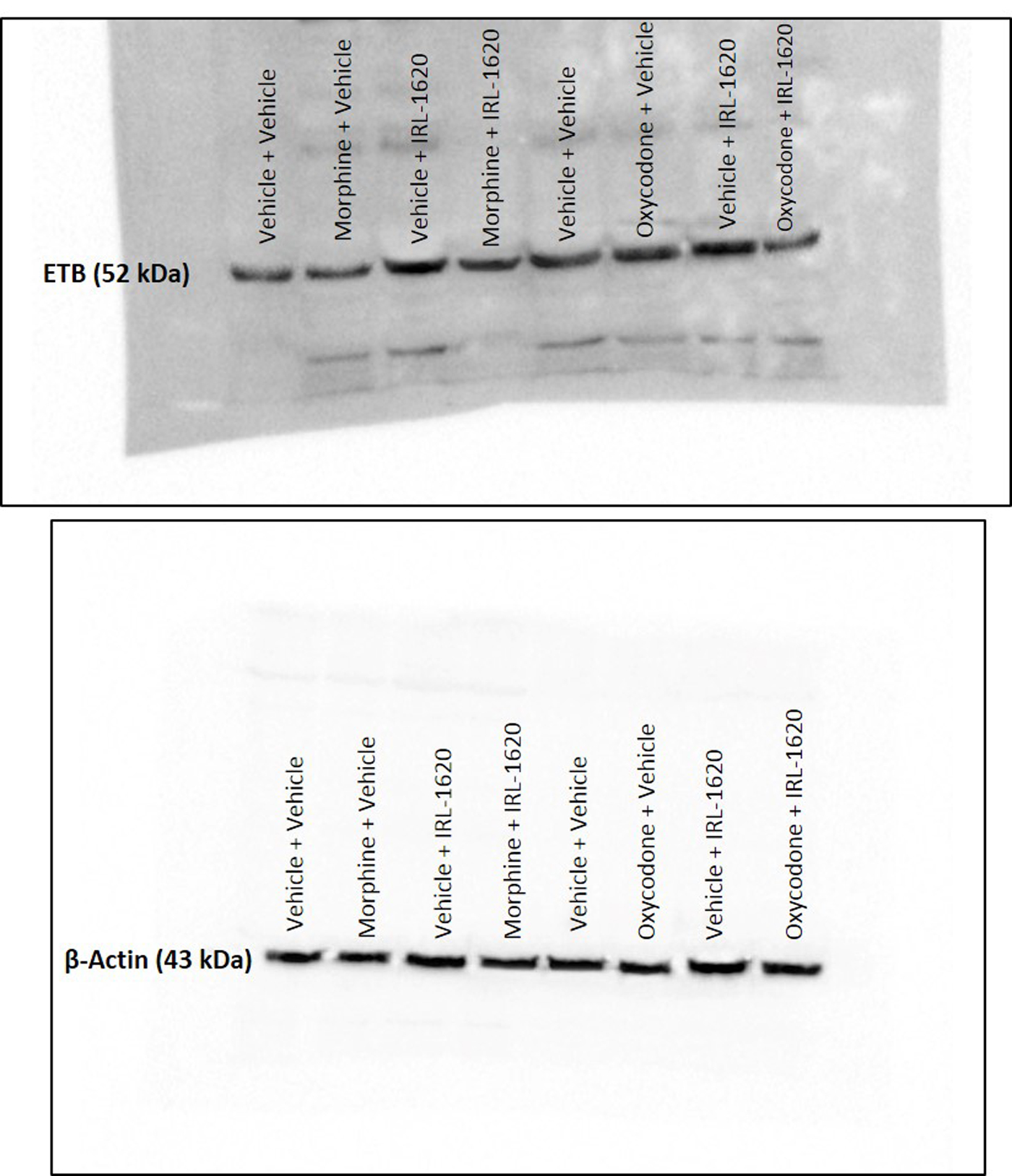

Supplement: Supplementary Fig. 5B [file mmc2.jpg]

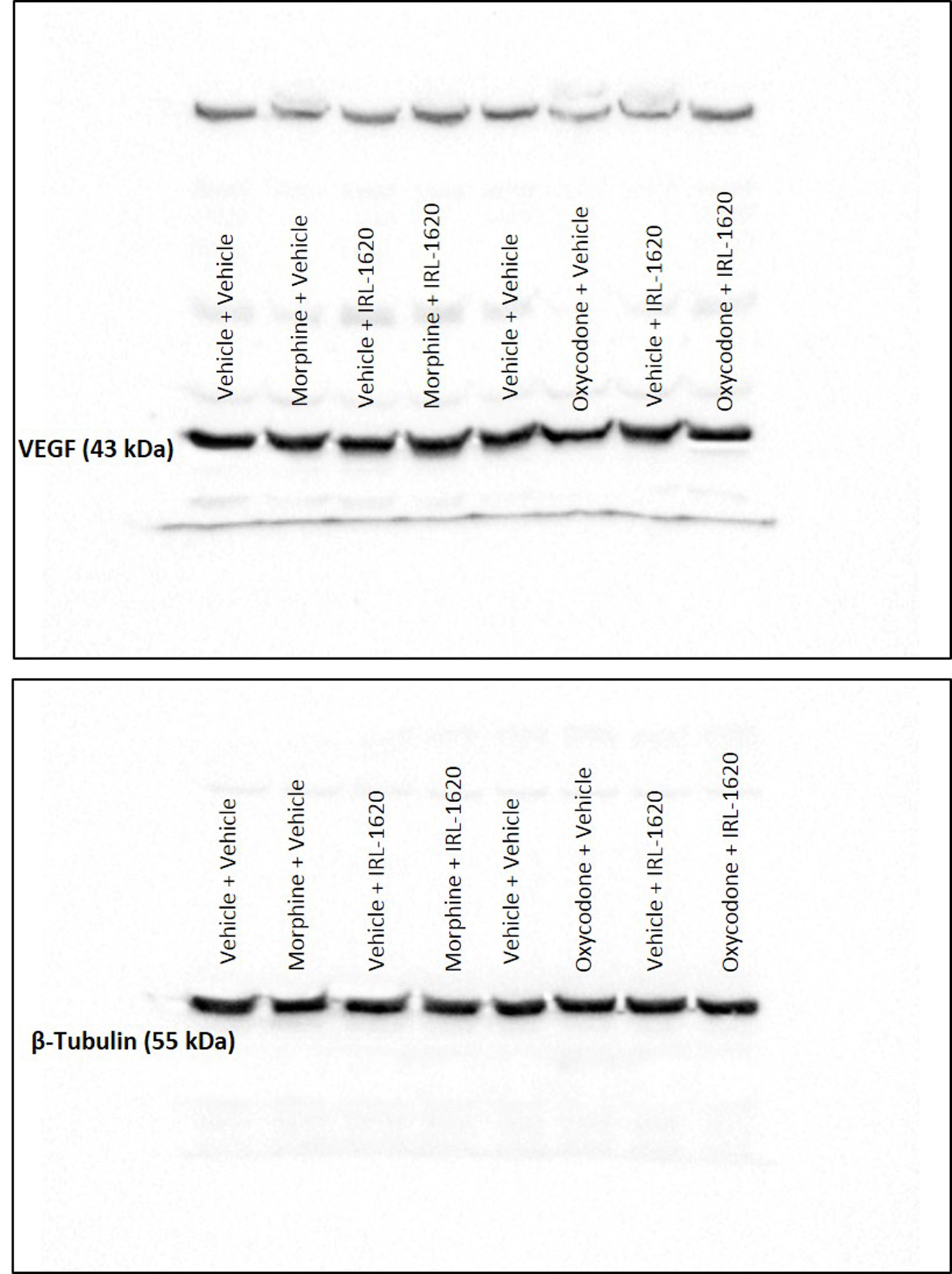

Supplement: Supplementary Fig. 6A [file mmc3.jpg]

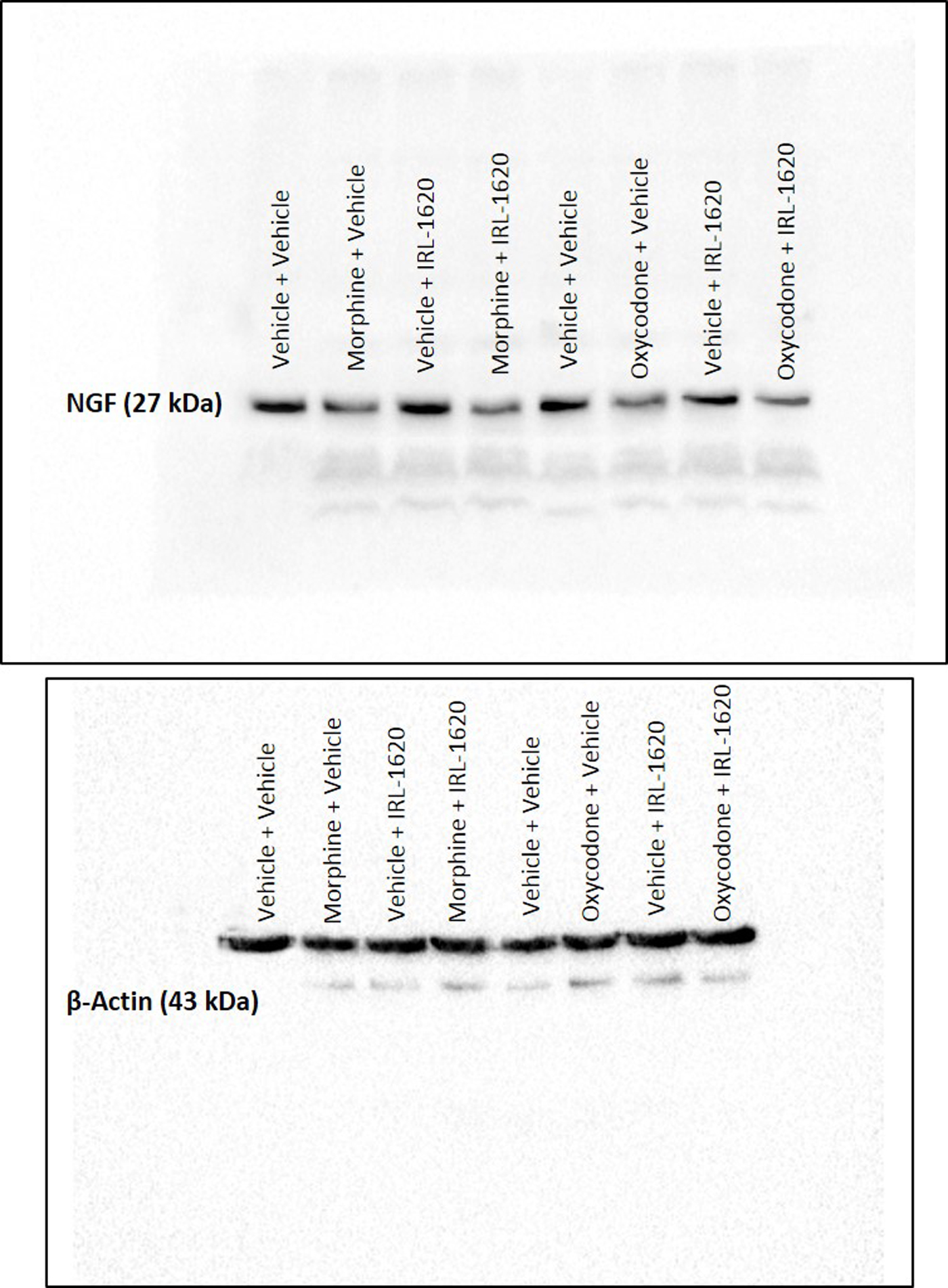

Supplement: Supplementary Fig. 6B [file mmc4.jpg]

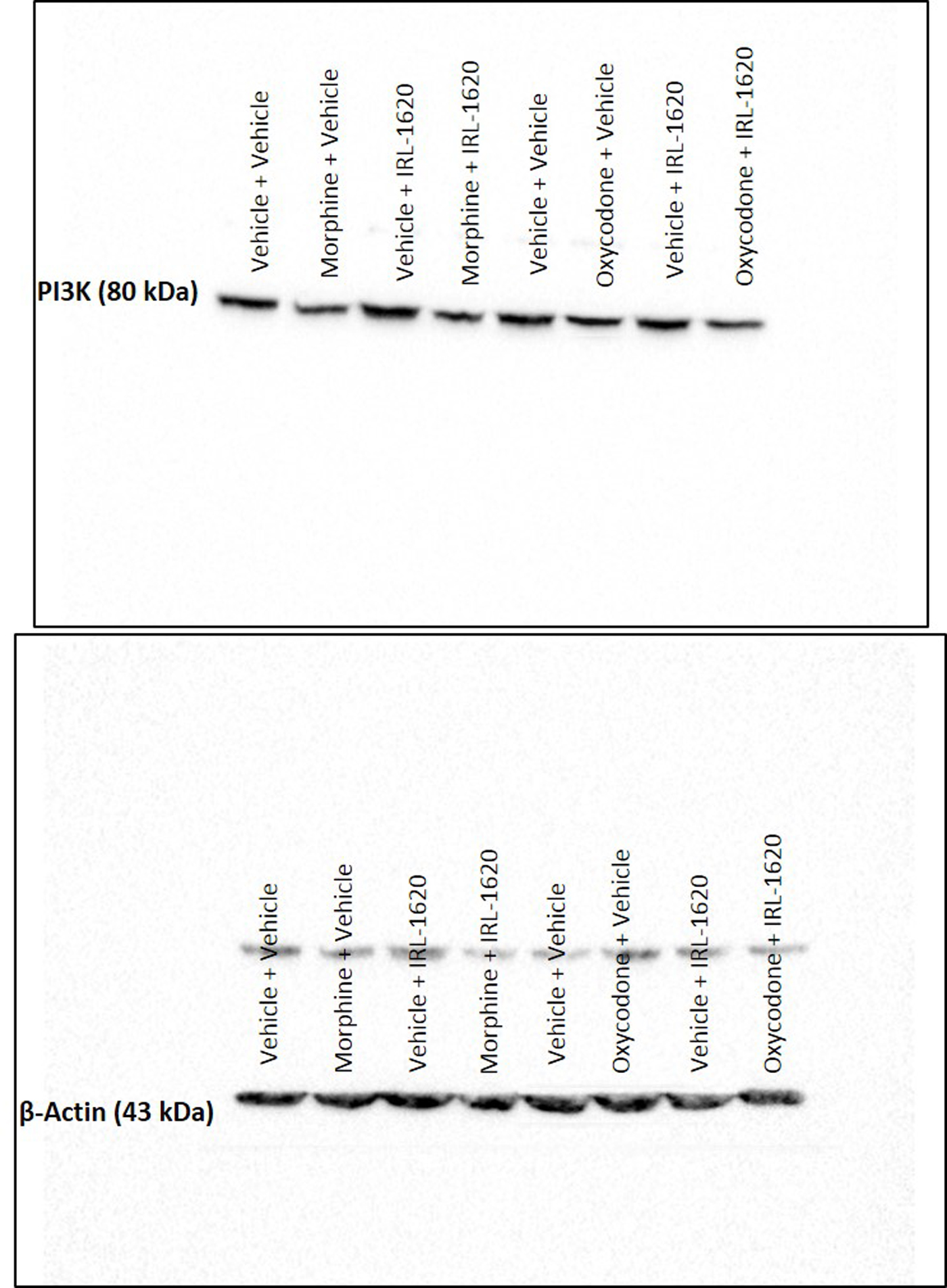

Supplement: Supplementary Fig. 7A [file mmc5.jpg]

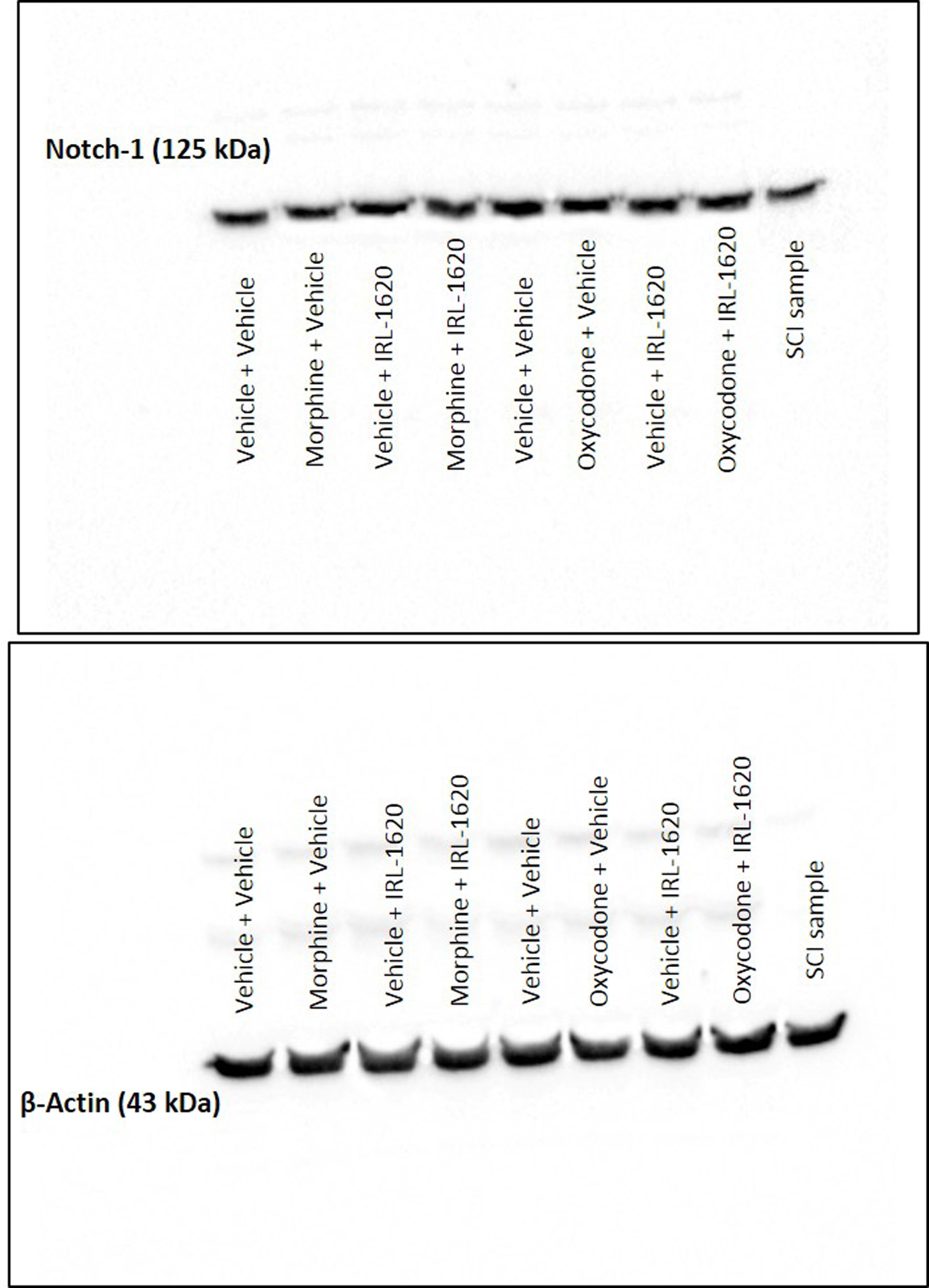

Supplement: Supplementary Fig. 7B [file mmc6.jpg]
